# Blocking VEGF by Bevacizumab Compromises Electrophysiological and Morphological Properties of Hippocampal Neurons

**DOI:** 10.3389/fncel.2019.00113

**Published:** 2019-03-26

**Authors:** Pauline Latzer, Olena Shchyglo, Tim Hartl, Veronika Matschke, Uwe Schlegel, Denise Manahan-Vaughan, Carsten Theiss

**Affiliations:** ^1^Department of Cytology, Institute of Anatomy, Ruhr University Bochum, Bochum, Germany; ^2^International Graduate School of Neuroscience, Ruhr University Bochum, Bochum, Germany; ^3^Department of Neurophysiology, Medical Faculty, Ruhr University Bochum, Bochum, Germany; ^4^Department of Neurology, Knappschaftskrankenhaus, Ruhr University Bochum, Bochum, Germany

**Keywords:** bevacizumab, LTP, neuronal function, spines, hippocampus

## Abstract

A hallmark of glioblastoma multiforme (GBM) is neoangiogenesis, mediated by the overexpression of vascular endothelial growth factor (VEGF). Anti-VEGF antibodies, like bevacizumab, prolong progression-free survival in GBM, however, this treatment has been reported to be associated with a decline in neurocognitive function. Therefore, this study focused on the effects of bevacizumab on neuronal function and plasticity. We analyzed neuronal membrane properties and synaptic plasticity in rat hippocampal slices, as well as spine dynamics in dissociated hippocampal neurons, to examine the impact of bevacizumab on hippocampal function and viability. VEGF inhibition resulted in profound impairments in hippocampal synaptic plasticity as well as reductions in dendritic spine number and length. Physiological properties of hippocampal neurons were also affected. These effects of VEGF blockade on hippocampal function may play a role in compromising memory and information processing and thus, may contribute to neurocognitive dysfunction in GBM patients treated with bevacizumab.

## Introduction

Glioblastoma multiforme (GBM) is the most aggressive primary brain tumor in humans, with a median post-diagnosis survival duration of patients of about 15 months (Stupp et al., 2009). GBM is characterized by a high degree of neovascularization mediated, among other mechanisms, by the overexpression of vascular endothelial growth factor (VEGF) in tumor cells (Plate et al., 1992; Lu-Emerson et al., 2015). VEGF is a dimeric polypeptide that binds to VEGF receptor 1 (VEGFR-1), VEGF receptor 2 (VEGFR-2) and the co-receptors neuropilin 1 and 2. The main effects of VEGF are mediated by VEGFR-2, including angiogenesis, endothelial cell proliferation and vascular permeability (Ferrara et al., 2003). The physiological role of VEGF is not restricted to the induction of angiogenesis. VEGF is expressed in the central nervous system by neurons, astrocytes and endothelial cells, where it exhibits neuroprotective and neurotrophic properties and stimulates neurogenesis (Matsuzaki et al., 2001; Jin et al., 2002; Rajah and Grammas, 2002; Rosenstein et al., 2003; Wang et al., 2005; Licht et al., 2010, 2011; Barouk et al., 2011). VEGF is also involved in neuronal function, as a transient exposure increases synaptic transmission in the hippocampus and an elevation of intracellular Ca^2+^ level and synaptic strength have been observed in hippocampal neurons (Kim et al., 2008; Yang et al., 2015). Moreover, VEGF plays a role in cognition: transgenic mice and rats that overexpress VEGF show improved contextual and spatial memory (Cao et al., 2004; Licht et al., 2011).

Inhibitors of the VEGF signaling pathway, such as bevacizumab, have been shown in several clinical studies to block angiogenesis to prevent tumor growth. Bevacizumab is a humanized monoclonal antibody against VEGF, which was granted approval by the US Food and Drug Administration authority in 2009 for its use in recurrent GBM (Presta et al., 1997; Gerber and Ferrara, 2005). Bevacizumab has also been tested in clinical trials for its therapeutic potential in newly diagnosed GBM supporting prolonged progression-free survival, but eliciting no overall effect on survival (Chinot et al., 2014; Gilbert et al., 2014). In addition, different cognitive tests conducted in patients treated with bevacizumab revealed cognitive deficits in some domains, such as oral word association, verbal learning and memory and mental flexibility (Gilbert et al., 2014). Besides this, intravitreal injection of bevacizumab increased apoptosis in retinal ganglion cells, and long-term incubation of neuronal cultures with bevacizumab exerted a cytotoxic effect on cortical cells and caused a decrease in hippocampal dendritic length (Romano et al., 2012; Latzer et al., 2016). Taken together, these data indicate that blocking VEGF prolongs progression-free survival in GBM patients, but may alter cognition, as well as neuronal morphology and function.

We addressed the question as to whether the cognitive deficits, that may accompany GBM treatment using the VEGF monoclonal antibody bevacizumab, may result from an impact on hippocampal function. Here, we focused on three specific aspects: (i) synaptic plasticity that is known to comprise a key component of information encoding resulting in associative memories; (ii) synaptic spines that are known to comprise the functional units of synaptic information encoding; and (iii) neuronal transmission/firing properties that are known to comprise the currency of information processing and relay, not only within the hippocampus, but in the brain as a whole (Kandel, 2001; Kemp and Manahan-Vaughan, 2004; Koch, 2004; Whitlock et al., 2006; Amtul and Atta-Ur-Rahman, 2015). Hippocampal slices were used to investigate long-term potentiation (LTP), which is a form of synaptic plasticity characterized by the strengthening of synaptic efficacy, as well as physiological properties of pyramidal cells in the hippocampus. In addition, spines dynamics were analyzed with the aid of live cell imaging of dissociated hippocampal cultures.

## Materials and Methods

The experiments were performed according to the European Communities Council Directive of September 22nd 2010 (2010/63/EU) for the care of laboratory animals with prior approval from the local ethics committee (Bezirksamt, Arnsberg, Northrhine Westphalia, Germany). Appropriate measures were taken to minimize animal pain and to reduce the number of animals.

### Drug Treatment

Bevacizumab (Avastin, B7106; Roche, Grenzach-Wyhlen, Germany) was applied at a concentration of 0.25 mg/ml to hippocampal slices and primary hippocampal neuronal cultures. First of all, this concentration allows the interaction of bevacizumab with human and mouse VEGF (Bock et al., 2007). In addition, 0.25 mg/ml is close to the plasma levels achieved in patients treated with 10 mg/kg of bevacizumab (von Baumgarten et al., 2011). Finally, 0.25 mg/ml of bevacizumab is in the range of concentrations widely used in previous studies investigating the effect of bevacizumab on different type of cells: bevacizumab has been applied in the range of 0–10 mg/ml in ocular cells (Spitzer et al., 2006), retinal ganglion cells (Iriyama et al., 2007; Brar et al., 2010), retinal Müller glial cells (Guo et al., 2010), corneal endothelial cells (Rusovici et al., 2011), human trabecular meshwork cells (Kahook and Ammar, 2010), retinoblastoma cells (Heo et al., 2012) and Schwann cells (Taiana et al., 2014) and a diversity of effects have been investigated. As a control, human IgG, Fc fragment antibody (Merck Millipore, AG 714) was applied at the same concentration (Foxton et al., 2013).

### Electrophysiology

#### Slice Preparation

Seven to 8-week-old male Wistar rats were anesthetized with isoflurane and then decapitated. Brains were dissected in ice-cold oxygenated medium containing (in mM): NaCl (87), KCl (2.4), NaH_2_PO_4_ (1.25), MgSO_4_ (1.3), CaCl_2_ (0.5), NaHCO_3_ (26) and D-glucose (2). Transverse hippocampal slices (400 μm thick) were prepared with a vibrating blade microtome (VTS1000, Leica, Germany) and slices were used for both field excitatory postsynaptic potential (fEPSP) recordings and for patch-clamp analysis. Immediately after cutting, slices were incubated for 30 min in a holding chamber with an oxygenated medium at 35°C. Then slices were placed on a nylon net in two submerged chambers with oxygenated artificial cerebrospinal fluid (aCSF) at 35°C containing (in mM): NaCl (124), KCl (3), NaH_2_PO_4_ (1.25), MgSO_4_ (1.3), CaCl_2_ (2.5), NaHCO_3_ (26) and D-glucose (13), to enable recording from two slices simultaneously (Scientific Systems Design Inc, Canada). Slices were continuously perfused with oxygenated aCSF (95% O2 and 5% CO2) at a constant flow rate of 1.5 ml/min. The temperature in the recording chambers was maintained at 30 ± 0, 5°C using the automatic temperature controller (PTC03; Scientific Systems Design INC, Canada). Slices were allowed to recover from cutting for 1 h before recording.

#### Whole Cell Patch Clamp

Patch clamp recordings were conducted as described previously (Novkovic et al., 2015b). Hippocampal slices were maintained at room temperature for 30 min before transfer to a recording chamber that was located on an upright microscope. Slices were continuously perfused with oxygenated aCSF (at a constant flow rate of 1–1.5 ml/min). Recording pipettes were pulled from borosilicate glass pipettes (1.5 mm external diameter) with a resistance of 6–14 MΩ and were then filled with intracellular solution (in mM: 97.5 potassium gluconate, 32.5 KCl, 5 EGTA, 10 Hepes, 1 MgCl_2_, 4 Na_2_ ATP, adjusted to pH 7.3 with KOH). Recordings were compensated for pipette capacitance and performed from visually determined cell bodies of pyramidal neurons in the CA1 *Stratum pyramidale*.

Whole-cell current-clamp recording was used to record intrinsic membrane properties. Recordings were subjected to low-pass filtering at 2.9 kHz and digitized at 10 kHz. FITMASTER software (HEKA, Lambrecht, Germany) and action potential (AP) feature (Matlab computer runtime) were used for offline analysis. The input resistance was deduced from the slope of the linear fit of the relationship between the change in membrane potential (ΔV) and the intensity of the injected current (between −60 pA and +20 pA). The time constant was determined from an exponential fit of the averaged voltage decay. During recording, the cells were maintained at their resting membrane potential. The mean of 15 s of basal recordings was used to determine the resting membrane potential. APs were generated by applying a square current (duration 600 ms) in the range from 5 pA to 300 pA with 5 pA steps. The minimum current needed to induce an AP was interpreted as the threshold current. The AP amplitude was measured as the voltage difference between the threshold and the peak of the potential. The AP peak reflected the maximal amplitude (in mV) of the AP. AP width was determined from the width at half-maximal amplitude (half-width) or at 20% of the maximal amplitude. Firing properties were examined by applying current steps of Δ50 pA, as hyperpolarizing and depolarizing square pulses (1-s duration) through the patch-clamp electrode (in the range of −300 pA to 400 pA). Bevacizumab and the human IgG, Fc fragment (control) antibody were applied using a closed circuit of aCSF (60 ml) containing 0.25 mg/ml bevacizumab (von Baumgarten et al., 2011) or control antibody. Slices were treated for 10 min with bevacizumab before recordings commenced.

#### LTP Study: Measurement of Evoked Potentials (Electrophysiological Recordings)

Procedures followed those used in previous studies (Novkovic et al., 2015a). A bipolar stimulation electrode (Frederik Haer, Bowdoinham, ME, USA) was positioned in the *Stratum radiatum* of the hippocampal CA1 region. Extracellular synaptic field potentials (fEPSPs) were recorded using a borosilicate glass-micropipette (1–2 MOhm) filled with aCSF. Biphasic test-pulse stimuli were applied (0.025 Hz and 0.2-ms duration) using a stimulus isolator. Evoked fEPSPs were recorded with a sample rate of 16,000 Hz. Five evoked responses were averaged for each time-point. Prior to commencing experiments, an input-output (I/O) relationship was determined to detect the maximum fEPSP obtainable (stimulation intensity applied in the range of 60–660 μA). A stimulation strength that evoked 50% of the maximum fEPSP observed in the input-output (I/O) assessment was used for test-pulse stimulation to generate basal synaptic responses (baseline). Following baseline recordings for 40 min, LTP was induced with high-frequency stimulation (HFS) consisting of 3 stimuli trains of 100 pulses at 5 min intervals. After LTP induction, synaptic responses were recorded for 2 h. Responses were amplified using a low pass filter at 1 kHz with (EXT-02F/2, npi electronics, Germany), and digitized for subsequent offline analysis (Micro 1401, CED Limited, England). Bevacizumab and the human IgG, Fc fragment (control) antibody were applied using a closed circulation of aCSF (60 ml) containing 0.25 mg/ml bevacizumab (Avastin, B7106; Roche, Grenzach-Wyhlen, Germany), or control antibody (Merck Millipore, AG714). When bevacizumab and control antibody were used, the fEPSP baseline was first recorded for 20 min, followed by a further 20 min of recordings in the presence of the drug, after which time HFS was applied.

### Dendritic Spine Analysis

#### Primary Dissociated Neuronal Culture

Primary dissociated neuronal cultures were obtained from Wistar rats at postnatal day 1. After decapitation, the hippocampi were dissected out of the cranium and into Hanks’ Balanced Salt solution (H8264; Sigma-Aldrich). Following removal of the meninges and blood vessels, tissues were trypsinated (0.05% trypsin Thermo Fischer Scientific, Darmstadt, Germany) for 5 min with a Teflon-covered magnetic stirring bar. The clear phase containing dissociated neurons was collected and transferred into minimal essential medium (MEM, M2279; Sigma-Aldrich, Schnelldorf, Germany) supplemented with 10% horse serum (S9135; Biochrom, Berlin, Germany), 2 mM L-glutamine (G7513; Sigma-Aldrich), and 1,000 U/ml penicillin + 0.1 ng/ml streptomycin (P0781; Sigma-Aldrich), to stop trypsin activity. The procedure was repeated four times for a total period of 20 min. The cell suspension was then centrifuged at 2,400 *g* for 10 min at room temperature and the pellet was re-suspended in 4 ml of MEM supplemented with 10% horse serum, 2 mM L-glutamine, and 1,000 U/ml penicillin + 0.1 ng/ml streptomycin. Pre-plating of the cells was done in a 6 cm plastic culture dish for 90 min at 37°C with 5% CO_2_ to eliminate glial cells. After pre-plating, the supernatant containing neuronal cells was collected and centrifuged at 2,400 *g* for 10 min. The pellet was discarded in neuronal medium containing neurobasal medium (NB, 10888-022; ThermoFischer Scientific) supplemented with 1× B27 Supplement (17504044; ThermoFischer Scientific), 2 mM L-glutamine (G7513; Sigma-Aldrich), and 1,000 U/ml penicillin + 0.1 ng/ml streptomycin (P0781; Sigma-Aldrich). After counting, 2,50,000 cells were plated on coverslips (ø 32 mm, 02R321-D; Kindler, Freiburg, Germany) pre-coated with collagen, and incubated at 37°C in a modified atmosphere of 5% CO_2_ in the air. Half of the medium was changed twice a week.

#### Transfection of Hippocampal Neurons

After 14 days *in vitro*, hippocampal neurons were transfected with Effectene Transfection Reagent kit (301425; Quiagen, Hilden, Germany). One microgram of Lifeact-red fluorescent protein (RFP) was mixed with a DNA-condensation buffer and the enhancer for 1 s. The mixture was incubated for 5 min at room temperature. Then, Effectene reagent was added and the solution was mixed for 10 s, followed by an incubation of 10 min at room temperature. Finally, 500 μl of neuronal medium completed the mixture, which was dispersed on a 3.5 cm coverslip.

#### Live Cell Imaging of Dendritic Spines

After transfection of hippocampal neurons with Lifeact-RFP, live cell imaging was performed on 15–20 day old *in vitro* cultures using a spinning disk confocal microscope (60× objective; Nikon Eclipse Ti; Nikon, Düsseldorf, Germany). Cultures were maintained at 37°C (Visiscope Live cell imaging system; Visitron System, Puchheim, Germany) and dendritic spines were first imaged for 10 min every 20 s to obtain a reference “baseline.” Then, bevacizumab, or human IgG, Fc fragment (control) antibody, was applied and the same areas were imaged again for 80 min every 20 s. Dendritic spines were analyzed with ImageJ by measuring the length and the number of spines every 5 min before and after treatment and compared to control. The results are expressed as a percentage of the baseline.

### Data Analysis

For fEPSP recordings, six hippocampal slices obtained from the brains of six rats were used for control antibody experiments and eight slices from five rats were used for the assessment of bevacizumab effects. For live cell imaging, at least five different experiments were conducted: 15 neurons and 44 spines were analyzed with bevacizumab, and 16 neurons and 33 spines were investigated for human IgG, Fc fragment antibody. For fEPSP recordings and live cell imaging of spines, each point shown in the graphs represents the mean value ± the standard error of the mean (SEM) relative to the baseline. To check for normal distribution, the chi-squared goodness-of-fit test was used, with rules of −2 and +2 for skewness and kurtosis. Statistical significance was estimated using a two-way analysis of variance (ANOVA) with repeated measures. A *post hoc* Student’s *t*-test was conducted to test for statistical significance of individual time-points. For patch clamp recordings, 22 neurons from six rats were recorded in control antibody experiments, and 22 neurons from seven rats were assessed in the presence of bevacizumab. Data were examined with a one-way ANOVA, except for the firing frequency experiments, where a two-way ANOVA was applied. For all statistical results, the significance level was set to *p* < 0.05.

## Results

### Some Active, but no Passive, Physiological Properties of Hippocampal Neurons Are Altered by Bevacizumab

We first explored if passive and active membrane properties of CA1 hippocampal neurons are affected by bevacizumab. Whole cell patch clamp recordings revealed that exposure to the VEGF antibody for periods as short as 10 min moderately affected pyramidal cell physiology compared to controls (*N* = 6, *n* = 22 for controls, *N* = 7, *n* = 22 for bevacizumab whereby “N” signifies the number of hippocampal slices, and “n” signifies the number of neurons).

**Figure 1:**
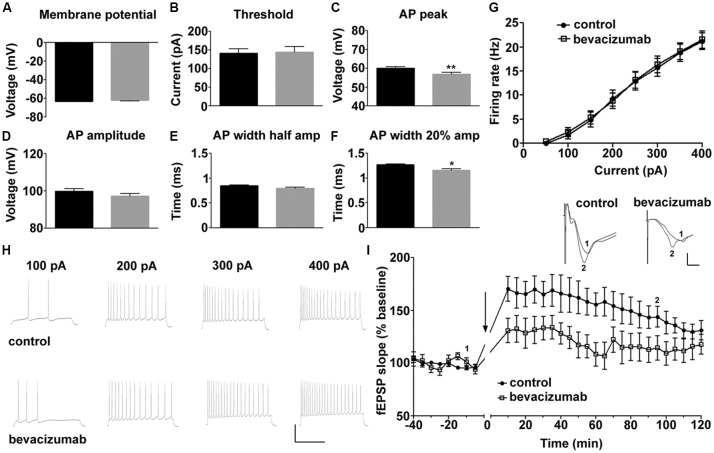
Bevacizumab alters some action potential (AP) properties of hippocampal pyramidal cells and impairs long-term potentiation (LTP). **(A)** The resting membrane potential is equivalent in bevazicumab-treated and control hippocampal slices. **(B–F)** In slices treated with bevacizumab, the current needed to trigger an AP was comparable to slices treated with control antibody **(B)**. However, the AP peak (amp; **C**) was significantly reduced in bevacizumab-treated hippocampus. Although the AP amplitude **(D)** and the width of the AP at half-maximal AP amplitude **(E)** were unaffected, the AP width at the 20% point **(F)** was decreased. **(A–F)** black = control; gray = bevacizumab. **(G)** Application of current steps (50–400 pA) revealed that firing rate of hippocampal neurons treated with bevacizumab is not significantly altered compared to controls. **(H)** Representative neuronal firing responses following the application of 100 pA, 200 pA, 300 pA and 400 pA of current. The vertical scale-bar corresponds to 50 mV and the horizontal scale-bar corresponds to 600 ms. **(I)** High-frequency stimulation (HFS) of Schaeffer collateral inputs to the CA1 region resulted in LTP that lasted for 2 h in hippocampal slices that were treated with control antibody. LTP in bevacizumab-treated slices was significantly impaired compared to LTP elicited in controls. The black line in the graph signifies application of bevacizumab in the corresponding experiment. Inset: representative analog traces showing fEPSPs that were evoked at the time-points indicated in control and bevacizumab-treated slices. The vertical scale-bar corresponds to 0.5 mV and the horizontal scale-bar corresponds to 2 ms. *P < 0.05; **P < 0.01.

Compared to slices that had been treated with control antibody (see “Materials and Methods” section), no significant changes were detected in resting membrane potential (Figure 1A), input resistance (Rin) or membrane time constant (Tau; Table 1, gray panels).

**Table 1:** Comparison of physiological properties of hippocampal neurons that were treated with bevazicumab or control antibody.

CA1 Pyramidal cells	Control antibody	Bevacizumab	*p*-value

Rin (MΩ)	96.7818182 ± 6.6051809	86.5714286 ± 5.4692088	*0.1219*
Tau (ms)	14.1031818 ± 0.75917158	13.5068182 ± 0.85336066	*0.4359*
RMP (mV)	−63.06 ± 0.5893	−61.85 ± 0.6923	*0.2989*
Threshold (pA)	141.1 ± 12.08	144.3 ± 14.48	0.7825
Threshold (mV)	−39.85865 ± 0.87458644	−40.4506682 ± 0.9737447	*0.6730*
AP amp (mV)	99.84 ± 1.385	97.27 ± 1.457	*0.1532*
AP peak (mV)	59.99 ± 0.8207	56.81 ± 1.122	0.0070**
AP time to peak (ms)	0.37727273 ± 0.00971859	0.37954545 ± 0.01458632	*0.9239*
AP peak to AHP (ms)	3.21590909 ± 0.38793195	3.23863636 ± 0.4538428	*0.4954*
AP total spike time (ms)	3.59318182 ± 0.39167881	3.61818182 ± 0.45888942	*0.6262*
AHP min (mV)	−47.9306682 ± 0.7448989	−47.6283727 ± 0.75713176	*0.8710*
AHP depth (mV)	8.07202273 ± 0.75777083	7.1777 ± 0.69012641	*0.5373*
AP width half amp (ms)	0.85012727 ± 0.0122762	0.77764545 ± 0.02389338	*0.0554*
AP width 20% amp (ms)	1.26803636 ± 0.0176279	1.17569091 ± 0.03679007	0.0124*

This table provides the mean values obtained for input resistance (Rin, in MΩ), membrane time constant (Tau, in ms), resting membrane potential (RMP, in mV; gray panels), threshold (in pA and mV), action potential (AP) amplitude, peak, time-to-peak, as well as the time from AP peak to after hyperpolarization (AHP), total spike duration, after hyperpolarization minimum and depth, and the width of the AP at the point where either 50% (half-width) or 20% of the maximal amplitude (amp) was attained (N = 6, n = 22, in all cases). Statistical significances of p-values are signified with an asterices: *P < 0.05, **P < 0.01.

Some features of the AP were affected, however (Figures 1B–F, Table 1). For example, although the current was required to elicit an AP was equivalent (Figure 1B), the AP peak (Figure 1C) was reduced, as was the width of the AP at the point of the AP where 20% of the maximal amplitude was attained (Figure 1F). The AP amplitude (Figure 1D) and the width of the AP at half-maximal spike amplitude (half-width; Figure 1E) were unaffected by bevacizumab (see Table 1 for statistical significances).

AP firing rate was equivalent in slices treated with bevacizumab and control antibody (Figures 1G,H) and membrane properties such as AP time-to-peak or the after-hyperpolarization were unaffected (Table 1). Thus, the effects of bevacizumab on pyramidal cell properties were relatively mild, whereupon small but nonetheless significant effects on AP peak and width were observed.

### Bevacizumab Prevents Both the Early and Later Phases of LTP

HFS of afferent fibers elicited LTP of CA1 synapses that lasted for at least 2 h in slices treated with control antibody or bevacizumab (Figure 1I; *N* = 6, *n* = 6 for controls, *N* = 8, *n* = 5 for bevacizumab whereby “N” signifies the number of hippocampal slices, and “n” signifies the number of animals). A significant impairment of LTP was evident throughout the entire recording period after HFS for slices treated with bevacizumab (ANOVA: *F*_(1,14)_ = 10.11; *p* = 0.0067). Here, both the early and later phases of LTP were affected. Five minutes after HFS, evoked responses were 131% of pre-HFS baseline values, compared to 170% at the same time-point in slices treated with control antibody (*t*-test, *p* = 0.0341; Figure 1I). One hour after HFS, evoked responses were 108% of pre-HFS baseline responses in comparison to 156% for control slices (*t*-test, *p* = 0.0102; Figure 1I).

### Spine Dynamics

**Figure 2:**
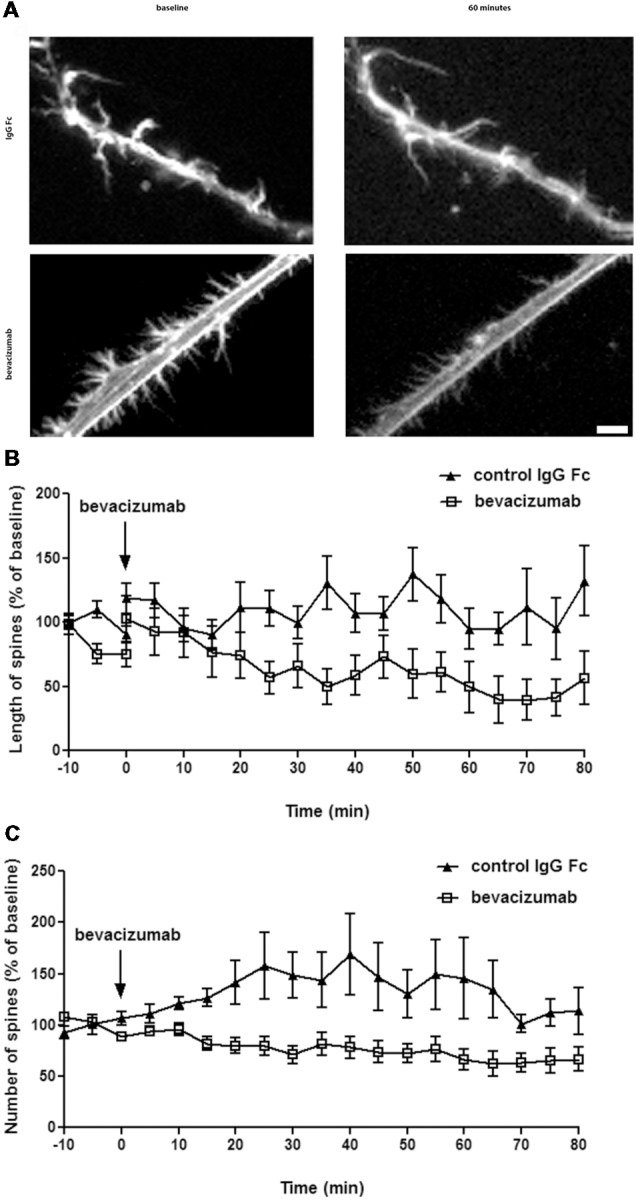
Spine length and density decrease after incubation with bevacizumab. **(A)** Live cell imaging was conducted in neuronal cultures that were treated with either bevacizumab or control antibody (*N* = 30 neurons, *n* = 90 spines). Dendritic spine images were generated using maximum projections of z-stacks (using ×60 objective). Data in line graphs represent the mean standard error of the mean (SEM). **(B)** Spine length is affected by bevacizumab. After 35 min of treatment, bevacizumab significantly decreased the length compared to control antibody-treated neurons. The spine length fluctuated around 1.2 μm. **(C)** Bevazicumab-treatment resulted in a decrease in the number of spines. The spine density fluctuated around six spines/10 μm of dendrites in controls and bevazicumab-treated cells.

To study the effect of bevacizumab on dendritic spines, we performed live cell imaging of hippocampal neurons. During a period of 10 min, the neurons were first imaged every 20 s to generate data that were then used as a reference “baseline.” Then nutrient medium, in the absence or presence of bevacizumab or control antibody, was added to the culture. Thereafter, the same neurons were imaged again for 80 min. We analyzed the length and the number of spines and found that bevacizumab significantly reduced the length of spines during the whole recording period (overall effect on spine length during entire recording period; ANOVA: *F*_(1,64)_ = 18.29; *p* < 0.0001; Figure 2A). More precisely, after 5 min the length of spines reached 117 ± 13% of the baseline for control neurons and only 51 ± 13% in presence of bevacizumab (*t*-test, *p* = 0.0006). After 60 min, spine length of control neurons attained 95 ± 16% of the baseline, whereas it had decreased to 31 ± 13% of the baseline when bevacizumab was applied (*t*-test, *p* = 0.0037; Figure 2B). In addition, bevacizumab significantly decreased the number of spines in comparison to treatment with control antibody (ANOVA: *F*_(1,38)_ = 8.45; *p* = 0.0061; Figure 2C). More specifically, after 5 min the number of spines reached 111 ± 9% of the baseline for control neurons and 63 ± 12% when bevacizumab was present (*t*-test, *p* = 0.0032). After 60 min, spine density of control neurons attained 146 ± 39% of the baseline, whereas it had decreased to 49 ± 11% of the baseline when bevacizumab was applied (*t*-test, *p* = 0.0246; Figure 2C). These data indicate that both the structure and the density of the spines are affected by bevacizumab after a short and long-term period of incubation.

## Discussion

In this study, we report for the first time that inhibition of the VEGF pathway profoundly affects hippocampal synaptic plasticity, neuronal responses and spine integrity. These effects were elicited by treatment of hippocampal tissue with bevacizumab, an antibody directed to VEGF that is used for the treatment of recurrent GBM. We showed that bevacizumab impairs hippocampal LTP and affects active membrane properties of CA1 neurons in hippocampal slices. In addition, it reduces hippocampal spine length and spine number in primary hippocampal neuronal cultures. These alterations may comprise the cellular basis for the reported effects of bevacizumab on cognitive function in GBM patients.

In general excitability, as reflected by AP firing properties and basal synaptic transmission at the level of the fEPSP were unaffected by bevacizumab. Another study also reported an absence of effects on synaptic transmission when blocking the VEGF signaling pathway (Ma et al., 2009). Here, Ca^2+^ influx, induced by glutamate or KCl application to hippocampal neuronal cultures, was analyzed by imaging and measuring Fluo-3 fluorescence intensity. Adding SU1498, a VEGFR-2 inhibitor did not affect either glutamate- or KCl-induced Ca^2+^ influx. As Ca^2+^ is necessary for neuronal function and communication, this study indicates that blocking VEGF signaling pathways does not affect these neuronal properties (Ma et al., 2009). In our study, we could not see any effect of bevacizumab on basal synaptic transmission. This suggests that bevacizumab does not influence Ca^2+^ influx in hippocampal neurons. In line with our findings, Huang et al. (2010) investigated mEPSC amplitude and frequency in cultured hippocampal neurons. Application of ZM322881 or SU1498 for 24 h to inhibit VEGFR-2 did not alter mEPSC properties. Altogether these data may indicate that endogenous VEGF does not play a role in synaptic transmission. This could be due to low endogenous VEGF levels in adult rat hippocampus (Inada et al., 2014).

In hippocampal slices, we observed that bevacizumab did not change passive membrane properties, but significantly reduced specific AP properties. In particular, the peak and the width of the AP were reduced. The width of the AP has been proposed to comprise an important determinant of the effectivity of inhibitory synchrony (Sato and Shiino, 2007) that is, in turn, an important component of neuronal oscillations and both cognitive and hippocampal information processing (Diba et al., 2014; Treviño, 2016; Schmidt et al., 2018). Smaller APs can be expected to have an impact on neurotransmitter release at the terminals (Südhof, 2013) and thus also affect information processing. Although both effects were not very pronounced, albeit significant, we observed potent debilitatory effects of bevacizumab on hippocampal LTP.

Here, both the initiation and the maintenance of LTP were impaired, suggesting that a permissive role for VEGF in signaling cascades required for the establishment of LTP. Similar results were obtained in transgenic rats expressing VEGFR-1 that binds and sequesters VEGF: in this case LTP could not be induced in the dentate gyrus (Licht et al., 2011). Prolonged treatment of rats with bevacizumab for a period of 30 days also resulted in an impairment of dentate gyrus LTP (Fathpour et al., 2014). Furthermore, an impairment of both VEGF-mediated synaptic potentiation (Kim et al., 2008) and of hippocampal LTP (De Rossi et al., 2016) was observed after pharmacological inhibition of the VEGFR2 receptor. Deficits in LTP *in vitro* were also detected in VEGFR-2 knock-out mice (De Rossi et al., 2016). Thus, inhibiting the VEGF signaling pathway either by blocking VEGFR or by targeting VEGF with bevacizumab, negatively affects LTP.

LTP is a key cellular mechanism for the generation of hippocampus-dependent memory that supports the encoding of specific aspects of spatial and context-dependent memory (Kemp and Manahan-Vaughan, 2004; Whitlock et al., 2006). Consequently, if LTP is impaired by blocking VEGF, memory is likely to be affected. Correspondingly, intra-hippocampal injection of PTK787/ZK222584, both VEGF blockers, in rats altered long-term spatial memory after 48 h (Pati et al., 2009). In addition, transgenic rats with mutated kinase insert domain protein receptors (*mKDR*) performed worse than control rats in passive avoidance tasks, and the presence of *mKDR* antagonized the enhanced spatial memory obtained with VEGF (Cao et al., 2004). Furthermore, the loss of VEGF function in transgenic mice reduces fear-conditioning memory (Licht et al., 2011). Thus, the decrease in LTP observed after bevacizumab treatment is a critical factor for neuronal function and synaptic plasticity and might predispose towards a decline in memory.

Synaptic plasticity is characterized by the rearrangement of neuronal structures such as axons and dendrites leading to a modification in the number and location of synapses (Amtul and Atta-Ur-Rahman, 2015). To check for structural rearrangements, implicated or resulting in the decrease in LTP that we observed in this study, we analyzed the length and the number of spines before and after treatment with bevacizumab. Five minutes after application of bevacizumab, spine length and number decreased and effects persisted over time. Our results are in accordance with other studies that investigated the effect of VEGF on spines. Diabetic mice, characterized by a loss of VEGF and VEGFR-2, express fewer spines in the hippocampus and show a dysfunction in memory compared to control mice (Taylor et al., 2015). Moreover, Licht et al. (2010) observed a decrease in spine density in newborn granule cells from the olfactory bulb of mice that exhibit a loss of function of VEGF. Furthermore, deletion of the hypoxia response element of the VEGF gene leads to the downregulation of several genes involved in the formation, maintenance and plasticity of synapses such as Apbb2, cadherin, Reelin, Fos, and CREB as well as pre-synaptic proteins involved in the release of neurotransmitter (Brockington et al., 2010). Thus, VEGF plays a crucial role in the foundation and maintenance of synapses. Furthermore, blocking VEGF with bevacizumab is detrimental to spines, an effect that in turn may contribute to the impairments of synaptic plasticity we observed in the present study.

On the structural level, actin filaments are enriched at the postsynaptic density of spines and regulate the shape and stability of the spine head and neck (Hering and Sheng, 2001). Moreover, actin dynamics are involved in spine formation and maintenance, synaptic adhesion, receptor endocytosis/exocytosis and in synaptic plasticity (Spence and Soderling, 2015). Therefore, in the present study, hippocampal neurons were transfected with a plasmid encoding for actin coupled to RFP so that spine dynamics could be scrutinized. Actin filaments are continually assembled and disassembled by several proteins/complexes (Maiti et al., 2015; Spence and Soderling, 2015). Here, we report a decrease in the length and in the density of spines over time following bevacizumab treatment that might be mediated through actin-related proteins. Actin and its related proteins are not only involved in the basal regulation of spines, but they are also implicated in synaptic plasticity. In fact, it has been shown that cofilin plays a role in facilitating structural LTP (Spence and Soderling, 2015). Moreover, CaMKII (calcium calmodulin kinase II), which is an important kinase involved in LTP, phosphorylates several actin-related proteins to induce spine morphological changes involved in synaptic plasticity (Hering and Sheng, 2001; Spence and Soderling, 2015; Woolfrey and Srivastava, 2016). Thus, as we observed both a decrease in LTP and spine dynamics with bevacizumab, we postulate that actin-related proteins are regulated by bevacizumab to mediate these effects. In addition, LTP induces the creation of new spines accompanied by an increase in spine size and synapse area (Hering and Sheng, 2001; Segal, 2005; Amtul and Atta-Ur-Rahman, 2015), and LTP stabilization is associated with changes in actin cytoskeleton and spine morphology (Hlushchenko et al., 2016). Therefore, it is very likely that bevacizumab affects actin directly, or through actin-binding proteins, and as a result decreases LTP, spine density and spine length.

Indeed, we would like to point out a specificity of the morphology of our cultured neurons that show numerous filipodia with variances in the baseline of spine number and length. Filopodia are very dynamic, long (2–20 μm) and thin (<0.3 μm) structures in developing dendrites but also in mature neurons (Yuste and Bonhoeffer, 2004). Young tissue has highly motile filopodia for the development of neuronal circuitry (Bonhoeffer and Yuste, 2002), which would indicate that our culture is not mature but still on development, probably because of the lack of surrounding cells usually found in tissue or *in vivo*. Filopodia of collateral type occur mostly in p3–p12 animals (Yuste and Bonhoeffer, 2004), which is in the same time range of our dissociated culture. Juvenile mice (P18 and P22) show a high distribution of long and thin spines on L5 pyramidal neurons in the cortex, which may correspond to the filopodia-like structure that we can observe after 15 days of culture (Mostany et al., 2013). A study investigating neuronal cells of juvenile (p25) WT mice and neurons from a Rett-syndrome animal model *in vitro* also showed numerous filopodia at this stage of age (Landi et al., 2011). In fact, the density of filopodia was even higher than spine density *in vivo*, which is in correlation with our observations. Filopodia serve as a precursor to spines, are involved in dendritic growth and branching in activity-independent manner as well as they are responsible for activity-dependent synaptogenesis. Even mature neurons have the machinery necessary for extensive spinogenesis. Furthermore, spine morphology changes continuously notably depending on activity. Filopodia actively search for presynaptic partners and become then protospines, immature spines and finally spines (Bonhoeffer and Yuste, 2002). It has also been shown that reduced synaptic activity converts mature spines into filopodia to compensate for the loss of synaptic input (Blanque et al., 2015). As our culture is composed of dissociated neurons that are dispersed over the coverslip, filopodia are intensively searching for partners that might not be in the surrounding environment explaining the high number of filopodia and the high motility. Filopodia in dissociated culture are less structural restricted and can move, whereas spines surrounded by neutropil cannot (Bonhoeffer and Yuste, 2002). Thus our neurons reflect what can be expected of neuronal cultures at this developmental stage.

Impoverishments of spine morphology and density could be detrimental for brain function and have been reported in several neurodegenerative disorders (Hering and Sheng, 2001). In fact, spine loss and abnormalities are characteristic of brain diseases as Parkinson’s disease or Rett syndrome (Maiti et al., 2015). Therefore, the reduction in spine length and density that we observed following bevacizumab treatment may reflect cellular changes in the hippocampus that reflect an impairment of hippocampal information processing.

In summary, we observed that bevacizumab decreases active neuronal properties, reduces LTP, and diminishes the number and length of spines in hippocampal neurons. We propose that the cellular mechanisms, through which bevacizumab decreases neuronal properties and spine dynamics, involves the dysregulation of actin and its related proteins, which in turn alters LTP. Taken together our data suggest that bevacizumab may not only compromise synaptic information *encoding* in the form of LTP, it compromises hippocampal information *processing* and relay. This can be expected to have a pronounced negative impact on both memory function (in the form of new learning) in individuals treated with VEGF inhibitors, but can also be expected to debilitate other forms of hippocampus-dependent cognition such as memory retrieval and information discrimination (Kesner, 2013).

## Data Availability

All datasets generated for this study are included in the manuscript.

## Author Contributions

PL, OS and DM-V conducted the synaptic plasticity study and analysis. TH, OS and DM-V conducted the patch clamp study and analysis. PL, VM, US and CT conducted the cell culture/microscopy components of the study. PL and OS contributed equally to the experimental workload (joint first authors). DM-V and CT contributed equally to all aspects of this study. CT, US and DM-V contributed to the concept and design of this study. The article was written by DM-V with contributions from all authors.

## Conflict of Interest Statement

The authors declare that the research was conducted in the absence of any commercial or financial relationships that could be construed as a potential conflict of interest.
